# Suboptimal Dosing of β-Blockers in Chronic Heart Failure: A Missed Opportunity?

**DOI:** 10.1097/JCN.0000000000000847

**Published:** 2021-07-28

**Authors:** Melanie McGinlay, Sam Straw, Rowenna Byrom-Goulthorp, Samuel D. Relton, John Gierula, Richard M. Cubbon, Mark T. Kearney, Klaus K. Witte

**Affiliations:** **Melanie McGinlay, RN** Heart Failure Nurse Specialist, Leeds Teaching Hospitals NHS Trust, United Kingdom.; **Sam Straw, MBChB** BHF Clinical Research Fellow, Leeds Institute of Cardiovascular and Metabolic Medicine, Leeds, United Kingdom.; **Rowenna Byrom-Goulthorp, RN** Research Nurse, Leeds Teaching Hospitals NHS Trust, United Kingdom.; **Samuel D. Relton, PhD** Senior Research Fellow, Leeds Institute of Health Sciences, University of Leeds, United Kingdom.; **John Gierula, PhD** NIHR Post-Doctoral Research Fellow, Leeds Institute of Cardiovascular and Metabolic Medicine.; **Richard M. Cubbon, PhD** Associate Professor and Consultant Cardiologist, Leeds Institute of Cardiovascular and Metabolic Medicine.; **Mark T. Kearney, MD** Professor and Consultant Cardiologist, Leeds Institute of Cardiovascular and Metabolic Medicine.; **Klaus K. Witte, MD** Associate Professor and Consultant Cardiologist, Leeds Institute of Cardiovascular and Metabolic Medicine.

**Keywords:** blood pressure, cardiovascular nursing, chronic heart failure, heart rate, β-blockers

## Abstract

The evidence base for the benefits of β-blockers in heart failure with reduced ejection fraction (HFrEF) suggests that higher doses are associated with better outcomes.

**Objectives:**

The aim of this study was to report the proportion of patients receiving optimized doses of β-blockers, outcomes, and factors associated with suboptimal dosing.

**Methods:**

This was a prospective cohort study of 390 patients with HFrEF undergoing clinical and echocardiography assessment at baseline and at 1 year.

**Results:**

Two hundred thirty-seven patients (61%) were receiving optimized doses (≥5-mg/d bisoprolol equivalent), 72 (18%) could not be up-titrated (because of heart rate < 60 beats/min or systolic blood pressure <100 mm Hg), and the remaining 81 (21%) should have been. Survival was similarly reduced in those who could not and should have been receiving 5 mg/d or greater, and patient factors did not explain the failure to attain optimized dosing.

**Conclusions:**

Many patients with HFrEF are not receiving optimal dosing of β-blockers, and in around half, there was no clear contraindication in terms of heart rate or blood pressure.

β-Adrenoceptor antagonists (β-blockers) reduce morbidity and mortality, and alongside inhibitors of the renin-angiotensin system are first line for the treatment of heart failure with reduced ejection fraction (HFrEF).^1^ In clinical practice, these medications are usually started at low doses, with subsequent dose titration aiming for those proven in clinical trials. However, rates of attainment of optimal dosing of β-blockers are consistently low in clinical practice, prospective observational studies,^2^ and contemporary clinical trials.^3^

Failure to achieve optimal doses is likely to be multifactorial and variable within cohorts, with factors including those that could be overcome and some that are fixed. Recognized clinical factors include baseline disease severity, comorbidity, medication side effects, and cognitive dysfunction, whereas nonclinical factors such as system failure, clinician inertia, non-adherence, health knowledge, attitude, and perception are less well-explored.^4^

## Aims

The aims of this analysis were, first, to report the proportion of patients receiving optimized doses of β-blockers from a real-world cohort of patients with HFrEF, divided by those who could not be up-titrated because of blood pressure or heart rate limitations and those who should have been up-titrated; second, to report the outcomes of patients who were or were not receiving optimal dosing; and, finally, to explore clinical and demographic factors associated with failure to attain optimal dosing.

## Methods

### Study Design

This was a prospective cohort study in unselected ambulatory patients with HFrEF with the a priori aim of describing contributors to outcomes.

### Setting

The study was undertaken in specialist heart failure clinics in 4 UK hospitals combining hospital and community care. Healthcare professionals included cardiologists specializing in heart failure, heart failure nurse specialists, and a cardiac physiologist.

### Participants

Between June 2006 and January 2009, consecutive patients were approached to participate; in total, 628 were recruited, and of these, 408 underwent clinical and echocardiography assessment at the time of enrolment. Further assessment was conducted after 1 year to assess for changes in medical therapy, symptoms, and left ventricular remodeling after initiation of disease-modifying agents. Inclusion required signs and symptoms of chronic heart failure for at least 3 months, being 18 years or older, and left ventricular ejection fraction (LVEF) of 45% or less on transthoracic echocardiogram, based upon guidelines for diagnostic and therapeutic criteria in place at the time.

### Variables and Data Sources

At the time of study recruitment, patient demographics, etiology of heart failure, medical history, and functional capacity according to New York Heart Association classification were recorded. At baseline and again at 1 year, we measured heart rate and blood pressure, performed 2-dimension echocardiography, measured left ventricular end-diastolic diameter and LVEF by Simpson's biplane method, and obtained venous blood samples. For the purposes of analysis, doses of angiotensin-converting enzyme inhibitor, β-blocker, and loop diuretic were reported as equivalent doses, relative to the maximum licensed doses of ramipril, bisoprolol, and furosemide, as previously published.^5^ All patients were registered with the UK Office of Population Censuses and Surveys, which provided details of the time of death, with final censorship occurring in November 2018.

### Definitions and Outcomes

We contrasted patients who were or were not receiving optimized dosing of β-blockers at 1 year (defined as ≥5-mg bisoprolol equivalent dose of β-blocker), dividing those who were not optimized according to whether they could not (because of either heart rate < 60 beats/min or systolic blood pressure < 100 mm Hg) or should have been up-titrated (absence of either of these features). We report the proportions of patients who were, could not, and should have been receiving optimized dosing of β-blockers, the clinical and demographic factors associated with failure to up-titrate dosage at 1 year, and association with outcomes.

### Statistics

All statistical analyses were performed using IBM SPSS Statistics version 26 (IBM Corporation, Armonk, New York). After demonstrating normality of distribution, continuous variables are expressed as mean (SD). Discrete variables are presented as number and percentages in parentheses. Patients receiving optimized dosing were compared with those who were not using χ^2^ for categorical variables and by Student *t* test for continuous variables. Kaplan-Meier curves were used to plot survival and compared with log-rank test. Multivariate analyses used Cox proportional hazards regression, and in all analyses, statistical significance was defined as *P* < .05.

### Ethical Considerations

Ethical approval was given by the Leeds West Research Ethics Committee (07/Q1205/17) and conducted in accordance with the principles outlined in the Declaration of Helsinki. All patients gave informed written consent for inclusion and long-term electronic follow-up.

## Results

**FIGURE 1:**
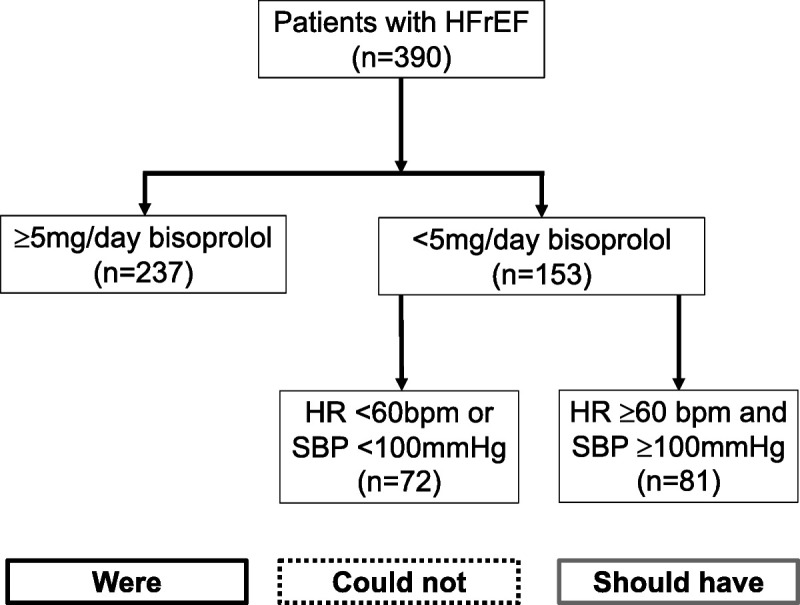
Study flowchart.

In total, 628 patients were recruited, and of these, 408 attended a follow-up visit at 1 year at Leeds Teaching Hospitals NHS Trust, 18 of which had missing data. Our final cohort consisted of 390 patients, of whom 295 (75.6%) were male with an average age of 66.4 (12.1) years (Table). Overall, 347 (85%) were prescribed a β-blocker (mean [SD] dose of 5.2 [3.7] mg/d), 237 (61%) were receiving optimized doses, 72 (18%) could not be up-titrated, whereas, on the basis of heart rate and blood pressure data, the remaining 81 (21%) should have been up-titrated but were not (Figure 1).

**TABLE:** Clinical Features at Baseline of Patients Who Could Not, Should Have, and Were Up-titrated to ≥5-mg Bisoprolol Equivalent Dose at 1 Year

	All Patients (N = 390)	≥5-mg Bisoprolol “Were” (n = 237)	<5-mg Bisoprolol “Could Not” (n = 72)	<5-mg Bisoprolol “Should Have” (n = 81)
Demographics				
Age, y, mean (SD)	66.4 (12.1)	64.3 (12.4)	69.6 (10.7)	69.7 (11.2)^a^
Male sex, n (%)	295 (75.6)	178 (75.1)	58 (80.6)	59 (72.8)
Medical history, n (%)				
Ischemic etiology	245 (62.8)	134 (56.5)	54 (75.0)^b^	57 (70.4)^a^
Diabetes mellitus	94 (24.1)	46 (19.4)	27 (37.5)^b^	21 (25.9)
COPD	38 (9.7)	13 (5.5)	8 (11.1)	17 (21.0)^c^
Pacemaker/defibrillator	138 (35.4)	90 (38.0)	17 (23.6)^a^	31 (38.3)
Observations, mean (SD)				
HR, bpm	72.5 (17.9)	72.8 (18.1)	68.6 (18.4)	75.1 (16.8)
SBP, mm Hg	121.7 (22.3)	121.7 (21.9)	113.1 (21.3)	128.7 (22.8)
NYHA class, n (%)				
I	82 (21.0)	60 (25.3)	8 (11.1)	14 (17.3)
II	172 (44.1)	103 (43.5)	33 (45.8)	36 (44.4)
III	129 (33.1)	71 (30.0)	29 (40.3)	29 (35.8)
IV	7 (1.8)	3 (1.3)	2 (2.8)	2 (2.5)
Medications, mean (SD)				
Ramipril equivalent dose, mg/d	5.1 (3.5)	5.2 (3.6)	4.9 (3.6)	4.6 (3.4)
Bisoprolol equivalent dose, mg/d	3.4 (3.0)	4.5 (3.0)	1.9 (2.1)^c^	1.6 (2.0)^c^
Furosemide equivalent dose, mg/d	54.1 (49.5)	51.9 (45.8)	63.6 (66.1)^b^	52.3 (43.2)
Laboratory investigations, mean (SD)				
Hemoglobin, g/dL	13.9 (1.8)	14.1 (1.9)	13.4 (1.6)	13.7 (1.8)
Creatinine, μmol/L	132.0 (67.6)	126.7 (50.0)	134.8 (51.5)	144.9 (110.8)^a^
Albumin, g/dL	42.9 (3.2)	43.2 (3.1)	42.4 (3.1)	42.5 (3.1)
Electrocardiogram, mean (SD)				
PR interval, ms	175.6 (37.2)	174.3 (31.1)	171.4 (32.6)	184.3 (53.0)^b^
QRS duration, ms	122.1 (30.1)	123.7 (31.5)	114.9 (25.6)^b^	124.0 (29.4)
Echocardiography, mean (SD)				
Baseline LVEF, %	30.8 (9.2)	30.2 (9.0)	31.9 (9.3)	31.2 (9.3)
Baseline LVEDd, mm	59.1 (9.2)	60.0 (9.2)	56.9 (8.8)	58.4 (8.9)

Abbreviations: bpm, beats per minute; COPD, chronic obstructive pulmonary disease; HR, heart rate; LVEDd, left ventricular end-diastolic diameter; LVEF, left ventricular ejection fraction; NYHA, New York Heart Association; SBP, systolic blood pressure.

^a^*P* < .05, ^b^*P* < .01, and ^c^*P* < .001 compared with ≥5-mg bisoprolol.

**FIGURE 2:**
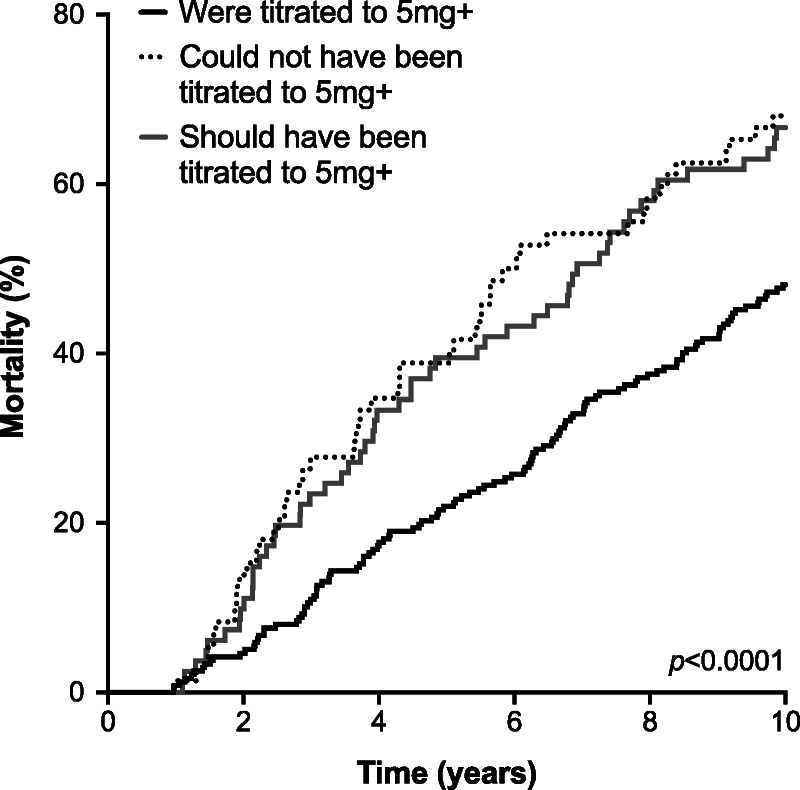
Kaplan-Meier plot of all-cause mortality divided by those who were, could not, and should have been receiving optimized dosing.

During a mean (SD) follow-up of 7.6 (3.4) years, there were a total of 242 deaths (59.3%). We observed clear stepwise benefits in longevity with those receiving the highest doses. When adjusted for age and sex, equivalent dosing of bisoprolol received at follow-up was associated with a reduction in mortality (heart rate, 0.95; 95% confidence interval, 0.91–0.98; *P* = .004), which persisted in multivariable analysis adjusted for differences between groups at baseline and follow-up (heart rate, 0.96; 95% confidence interval, 0.92–1.00; *P* = .029). Survival was lower in patients receiving suboptimal doses of β-blockers, regardless of whether they could not have been or should have been up-titrated (Figure 2).

Compared with patients receiving optimal therapy, patients who could not be up-titrated because of heart rate or blood pressure limitations were on average older, with comorbid ischemic heart disease or diabetes mellitus (Table). They were prescribed higher doses of loop diuretics compared with patients who were up-titrated and were less likely to be implanted with device therapy. Similarly, patients who were not up-titrated but could have been were older and more often had ischemic heart disease compared with those who were. The prevalence of chronic obstructive pulmonary disease was around 4 times higher than in patients who were up-titrated, with more renal impairment.

On the other hand, aside from older age, the 81 patients who should have been up-titrated had evidence of less severe heart failure at baseline, with better symptomatic status, higher systolic blood pressure, a lower rate of diabetes mellitus, and a higher rate of device implantation compared with patients who could not be up-titrated. However, the survival curves of these 2 groups were similar. For most patients who were not up-titrated but who should have been, baseline characteristics did not explain the failure to optimize therapy.

## Discussion

In this analysis, we have shown that, despite closely integrated hospital and community care multidisciplinary follow-up programs, in a real-world cohort of patients with HFrEF, approximately 40% were not receiving optimal doses of β-blockers 1 year after their first attendance. Furthermore, in around half of these, there were no objective contraindications, and despite similar or less severe heart failure by conventional measures at baseline, these patients were at a higher risk of adverse outcomes. Baseline characteristics did not explain failure to optimize doses of β-blockers for the majority suggesting that unmeasured or underexplored patient factors might be relevant to the effort to optimize therapies for patients with HFrEF.

Treatment guidelines recommending the use of β-blockers in HFrEF^1^ can draw upon data from multiple randomized controlled trials demonstrating improvements in outcomes.^6^ The strongest benefits to patients in terms of left ventricular remodeling, reducing hospitalizations, and extending longevity are observed in those receiving evidence-based doses,^7,8^ contrasting the less clear-cut advantages for those receiving higher doses of inhibitors of the renin-angiotensin system.^9–11^ In our study, not all patients received a β-blocker and the dosing was lower than is recommended; however, it was broadly in line with other contemporary registry studies, and we were able to distinguish those who “should have” or “could not” have been up-titrated.^12–14^ Optimal treatment of HFrEF includes pharmacological and device therapies with considerable cost implications, yet our data show that inexpensive and proven therapies are poorly applied. In our cohort, baseline patient factors failed to explain suboptimal dosing for most patients where heart rate and blood pressure were not limitations.

Patients with HFrEF who have comorbidities are at an increased risk of adverse outcomes, including sudden cardiac death, and derive additional protection from disease-modifying agents.^15^ Despite this, patients with comorbidities, especially obstructive pulmonary disease, are often prescribed lower doses of β-blockers, despite evidence that these medications are effective and can be safely administered.^16^

Nontargeted strategies to optimize medication doses such as additional nurse support or education can be effective but have considerable cost implications.^17^ However, targeted intervention, applied early in the care pathway, could improve the uptake of higher doses, which could have significant benefits to patients with minimal additional cost. Delivering targeted intervention requires identifying those at risk of suboptimal dosing, who have the potential to be up-titrated. We were unable to explain why most patients were not up-titrated. This failure to optimize therapy in the setting of closely integrated hospital and community care services raises the possibility that unmeasured and largely underexplored patient-related factors such as attitude, perceptions, beliefs, and knowledge might be relevant. The presence of mild cognitive dysfunction is also a common finding in patients with heart failure,^18^ which increases vulnerability to intentional or unintentional nonadherence.^19^

Knowledge about heart failure can be a key determinant of health behavior. Multidisciplinary heart failure clinics often include education as an intervention, and although education alongside more intensive follow-up can lead to changes in self-care behavior, they have a variable effect on hospitalization and healthcare utilization.^20–22^ There are currently no studies of education programs that have undertaken a previous assessment of patients' knowledge or perception of their condition, and therefore, none provides individualized education tailored to the patient-specific deficiencies of knowledge, possibly because the tools most commonly used do not allow for this level of reliability. In addition, no study authors have explored the improvement in knowledge of heart failure after an education intervention.^23,24^ Untargeted strategies to optimize medication doses are therefore costly with an uncertain benefit.

Targeting requires information on who, when, and what. Specifically, for an educational intervention to have the greatest possible change of success, perhaps we need to identify early after diagnosis which patients is unlikely to achieve or maintain optimizing treatment at 1 year despite being suitable. We also need to know the optimal time to provide an education intervention or additional community support. Although it is logical to provide this early on, patients might be more receptive once they have come to terms with a new diagnosis. We need to establish which aspects of knowledge are missing in an individual. Finally, we also need to understand the influence of early cognitive dysfunction on knowledge and learned behavior in this setting.

### Limitations

This was a carefully characterized cohort of patients with long-term electronic follow-up. The exclusion of patients with LVEF greater than 45% means our findings are not generalizable to those with preserved ejection fraction. Although the mechanism of action of β-blockers extends beyond heart rate and blood pressure, these are the barriers to up-titration nurses and physicians are most likely to encounter in clinical practice. The present analysis did not explore the impact of socioeconomic status; however, we have previously shown that much of the attributable risk of hospitalization and mortality from socioeconomic status relates to noncardiovascular events.^25^

## Conclusions

Despite carefully integrated hospital and community care, approximately 40% of patients with HFrEF did not receive optimal dosing of β-blockers, and in approximately 20%, this was not due to bradycardia or hypotension. These patients had worse outcomes, regardless of whether they should have been or could not be up-titrated. For most of these patients, we were unable to explain the reasons for suboptimal dosing suggesting that gaining an awareness of potentially underexplored patient factors such as disease knowledge and cognition could help healthcare professionals identify those at the highest risk, with targeted education and community support to facilitate better uptake of β-blockers.

**What’s New and Important**

- Many patients with HFrEF do not receive optimized doses of β-blockers, and half of these “should have” according to heart rate and blood pressure.
- Patients who “should have” been up-titrated have worse outcomes, similar to those who “could not” be up-titrated.
- Clinical characteristics do not explain this failure to optimize dosing, suggesting unmeasured and underexplored factors may be relevant.
